# Optimizing controlled-release urea and urea combinations for sustainable rice-wheat production under nitrogen reduction

**DOI:** 10.3389/fpls.2025.1576049

**Published:** 2025-06-26

**Authors:** Yu Tao, Dehai Zhang, Zhipeng Xing, Chuan Ni, Miao Ye, Zujian Zhang

**Affiliations:** ^1^ College of Agriculture, Yangzhou University, Yangzhou, China; ^2^ State Key Laboratory of Crop Genetics and Physiology of Jiangsu Province, Yangzhou University, Yangzhou, China; ^3^ Co-Innovation Center of Modern Industrial Technology of Grain Crops of Jiangsu Province, Yangzhou, China; ^4^ Research Institute of Rice Industrial Engineering Technology, Yangzhou, China

**Keywords:** controlled-release nitrogen fertilizer mode, nitrogen reduction, rice-wheat yield, nitrogen use efficiency, soil fertility, sustainable fertilization strategies

## Abstract

Excessive nitrogen fertilization poses a threat to agricultural sustainability. Achieving high crop yield while improving soil health under reduced nitrogen (N) input is a global concern. This two-year rice-wheat rotation study was designed with two N fertilizer levels (conventional N level and reduced N level) and four controlled-release urea (CRU) application modes, along with a control CK (farmer’s conventional fertilization mode). Effects on yield, nitrogen use efficiency (NUE), and soil fertility were compared. Polymer-coated urea (PCU) was employed as the CRU material in this study. Significantly lower annual yields were observed under sole PCU. The PCU + U treatments increased yields by 9.16–15.00% over CK. The highest rice-wheat yield and nitrogen recovery efficiency occurred with 40% PCU + 30% U basal fertilizer + 30% U panicle fertilizer application mode. Nitrogen reduction decreased yield and plant nitrogen but improved nitrogen recovery efficiency. However, the RN-PCU + U mode achieved yields comparable to CK. RN-PCU + U treatments provided stable yields and higher economic benefits, with the latter increasing by 6.3-35.7% compared to CK. Soil nutrient content varied significantly among PCU application modes. The RN 70% PCU + 30% U basal fertilizer showed the best performance on soil fertility: organic matter content was 2.9% higher than CK, and total nitrogen was 9.3% higher than CK after two rice-wheat rotations. Panicle fertilizer enhanced nutrient uptake and reduced residual soil nutrient levels. Correlation analysis indicated that increased plant N and soil available phosphorus were associated with higher yields, whereas elevated soil alkali-hydrolyzed N content promoted organic matter accumulation. PCU + U application can compensate for the decrease of rice-wheat yield under nitrogen reduction and improve the activity of soil nutrients. Our results suggest that the 70% PCU + 30% U basal application mode optimizes nitrogen efficiency, sustains rice-wheat yields, and enhances soil fertility under reduced nitrogen input. These findings could inform future sustainable agricultural practices designed to reduce nitrogen use while maintaining crop yield.

## 1 Introduction

Rice (*Oryza sativa L.*) and wheat (*Triticum aestivum L.*) provide essential nutrition to over half of the global population (FAO, 2023). In China, rice and wheat account for 24.89% and 19.87% of the total grain planting area, respectively, with their yields contributing 30.37% and 20.06% of the total grain production (China, 2024). Additionally, the rice-wheat rotation system is widely practiced in China, covering more than 5.92% of the total grain planting area, primarily in the middle and lower reaches of the Yangtze River (Yang et al., 2008). Thus, rice and wheat cultivation, along with the rice-wheat rotation system, plays a pivotal role in ensuring China’s food security and agricultural sustainability.

Rice and wheat growth is highly responsive to nitrogen (N) fertilization, with optimal N management being critical for achieving high yields in these staple crops (Ercoli et al., 2013; Nallanthighal et al., 2024; Xu et al., 2023). However, N fertilizer application is often accompanied by significant N losses through pathways such as volatilization, denitrification, and leaching. These losses substantially reduce N fertilizer efficiency, leading to low nitrogen use efficiency (NUE) in grain production. Studies indicate that low NUE is a critical factor undermining the sustainability of crop production (Zhang et al., 2015; Zhang X. et al., 2022). In China, the NUE of major grain crops is only 41.3%, far below the 50%-60% observed in developed countries (Li, 2024). Moreover, excessive N application not only reduces NUE but also exacerbates environmental issues such as water eutrophication, soil acidification, and greenhouse gas emissions (Gao and Cabrera, 2023; Wood et al., 2023; Zhang et al., 2015; Zhao et al., 2024). Therefore, improving NUE and minimizing N losses are urgent priorities for global agricultural sustainability.

To address this issue, controlled-release urea (CRU) has gained widespread attention and application as a novel fertilizer in recent years. By modulating the release rate of N, CRU helps reduce potential N losses during application, thereby enhancing NUE. Research shows that CRU effectively mitigates N volatilization and denitrification losses while ensuring a stable N supply for crops (Yuan et al., 2016; Zhang et al., 2018, 2024; Zhou et al., 2018). Additionally, CRU reduces fertilization frequency, saves labor, and has minimal environmental impact. However, CRU also has limitations. For instance, its prolonged release cycle and slow release rate may delay nutrient supply during certain growth stages, potentially affecting crop growth and yield (Ke et al., 2017; Zhang et al., 2021). This limitation can be effectively addressed through a strategic combination with conventional urea. Urea (U) provides immediate nitrogen availability to bridge critical growth stages (Ding et al., 2023; Shi et al., 2025; Tao et al., 2024), while CRU ensures a sustained supply during subsequent development phases, thereby synergistically improving crop production.

Existing studies primarily focus on the effects of combined CRU and conventional urea application in single-season crops. Zhang et al. (2024) conducted a meta-analysis revealing that when CRU constitutes 70% of the total N fertilizer, NUE and yield significantly improve in crops like rice and wheat. Ke et al. (2017) demonstrated that combined CRU and urea application effectively reduces soil ammonia volatilization while enhancing rice yield and NUE. Zheng et al. (2016) found that in a wheat-maize rotation system, a 7:3 ratio of CRU to urea improved soil pH, maintained soil fertility, and promoted sustainable high yields. However, most studies concentrate on single-season crops, and research on the long-term effects and mechanisms of combined CRU and urea application in rice-wheat rotation systems remains limited. Particularly under reduced N fertilization, the impact of combined CRU and urea application on the yield and soil fertility of rice-wheat rotation systems has not been thoroughly explored. Therefore, investigating the long-term effects and optimal application modes of combined CRU and urea in rice-wheat rotation systems is crucial for improving crop yield, NUE and ensuring sustainable agriculture.

This experiment selected Jinxiangyu 1 (rice) and Yangmai 23 (wheat) as test materials, using polymer-coated urea (PCU) as the CRU material. A two-year rice-wheat rotation was conducted under varying N levels and PCU application modes. The aims were: (1) to evaluate the effects of different PCU application modes on annual yield formation, N recovery efficiency, and soil fertility in the rice-wheat rotation system; and (2) to identify which PCU application mode could offset the negative impacts of yield reduction under reduced N fertilization and elucidate its mechanisms. The ultimate goal was to identify the optimal PCU application mode for rice-wheat rotation systems, achieving high yields and efficiency under reduced N fertilization.

## 2 Materials and methods

### 2.1 Site description

**Figure 1:**
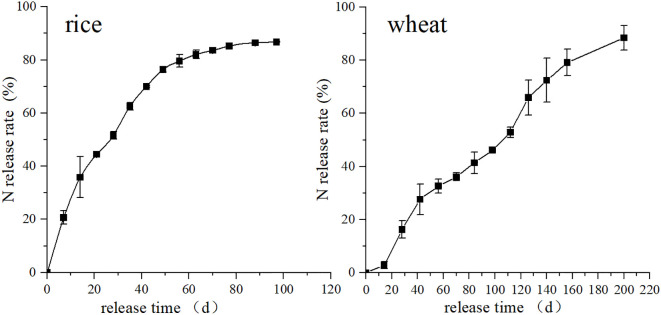
Accumulation rate of release of PCU in the fields over the rice and wheat season. The release of PCU is measured once every 7 days in rice season and once every 14 days in wheat season.

The experimental site was located in Xinmingzhou (32°16″ N, 119°34″ E) in Zhenjiang, Jiangsu Province, China. This study was conducted a continuous rice-wheat rotation experiment at the same location from 2021 to 2023. The rice variety used for testing was Jinxiangyu 1, while the wheat variety was Yangmai 23. Both were widely cultivated in the middle and lower regions of the Yangtze River. The experimental soil was classified as sandy loam. The top-layer soil (0–20 cm) at the experimental site had a pH of 7.96 (1: 2.5, soil/water), organic matter content of 28.2 g kg^−1^, total N content of 1.92 g kg^−1^, alkali-hydrolyzable N content of 143.86 mg kg^−1^, total P content of 1.02 g kg^−1^, Olsen-P content of 40.67 mg kg^−1^, total K content of 23.2 g kg^−1^ and an NH_4_OAc-exchangeable K content of 105.49 mg kg^−1^. The region has a perennial average annual temperature of 16.2°C and an average annual rainfall of 729.35 mm. PCU (N =44.5% N) was provided by Maoshi Agricultural Technology Co. Ltd., Anhui, China. The release rate of PCU was shown in **Figure 1**. After the application of base fertilizer, 40 net bags with 10 g PCU were buried in the field. Fertilizer pellets were collected from three mesh bags at 7-day (rice season) and 14-day (wheat season) intervals. Samples were homogenized in water, ground, and diluted to 0.5 L. The concentration of these solutions was measured using the Kjeldahl method (Douglas et al., 1980), and the release amount was calculated to plot the fertilizer release curve.

### 2.2 Experimental design and treatments

Each experimental plot area was 600 m^2^ (60m × 10m), with each treatment replicated three times. This study was conducted using a split-plot design. The main experimental factors were nitrogen levels (conventional nitrogen level (CN) and reduced nitrogen level (RN). The sub-experimental factors included four different CRU application modes of CRU combined with U (P1, P2, P3, and P4). The sub-factors were randomly assigned to subplots within each main treatment. The CK was applied under the conventional nitrogen level (CN). Additionally, a control group (CK) was established, which followed the local conventional fertilization practice, involving the application of urea only. (**Table 1**). In the CK treatment, the total nitrogen application during the entire growth period of rice was 300 kg hm^-2^, distributed as follows: basal fertilizer, tillering fertilizer, and at the stages of the 4th and 2nd last leaves in a ratio of 7:7:3:3, and that of wheat was 240 kg hm^-2^, distributed in a 2:1:1 ratio among basal fertilizer, return green fertilizer, and panicle fertilizer (3rd last leaf). Total N inputs were 300 kg hm^-2^ (rice) and 240 kg hm^-2^ (wheat) under CN, and 240 kg hm^-2^ (rice) and 180 kg hm^-2^ (wheat) under RN. In this experiment, polymer-coated urea (PCU) was used as CRU material. Four types of CRU application modes were tested. Treatment P1 was basal fertilizer application of 100% PCU; treatment P2 was basal fertilizer application of 70% PCU + 30%U; treatment P3 was basal fertilizer application of 70% PCU and panicle fertilizer application of 30%U; treatment P4 was basal fertilizer application of 40% PCU + 30%U and panicle fertilizer application of 30%U. The wheat treatment was consistent with the rice treatment, and all treatments were applied in the same field every season. All treatments received uniform phosphorus (P) and potassium (K) fertilization at an N: P_2_O_5_: K_2_O ratio of 2:1:1.2. Additionally, we established a zero-nitrogen (0N) control treatment. Due to significantly larger data discrepancies between the 0N treatment and other treatments, it was excluded from statistical analysis and is presented as reference data only. The 0N treatment results were: 5.66 t hm^-2^ rice yield with 82.57 kg hm^-2^ plant N, and 2.06 t hm^-2^ wheat yield with 62.00 kg hm^-2^ plant N in 2021; 6.20 t hm^-2^ rice yield with 72.03 kg hm^-2^ plant N, and 2.25 t hm^-2^ wheat yield with 75 kg hm^-2^ plant N in 2022.

**Table 1:** Experimental design of nitrogen fertilizer application modes in rice-wheat rotation system.

N	M	Rice season N (kg hm^-2^)							Wheat season N (kg hm^-2^)					Annual N (kg hm^-2^)
		Total N	Basal fertilizer		Tillering fertilizer	Panicle fertilizer			Total N	Basal fertilizer		Return green fertilizer	Panicle fertilizer 3^rd^ last leaf	Total N
			PCU	U		4^th^ last leaf	3^rd^ last leaf	2^nd^ last leaf		PCU	U			
CK		300	–	35%	35%	15%		15%	240	–	50%	25%	25%	540
CN	P1	300	100%						240	100%				540
	P2	300	70%	30%					240	70%	30%			540
	P3	300	70%				30%		240	70%			30%	540
	P4	300	40%	30%			30%		240	40%	30%		30%	540
RN	P1	240	100%						180	100%				420
	P2	240	70%	30%					180	70%	30%			420
	P3	240	70%				30%		180	70%			30%	420
	P4	240	40%	30%			30%		180	40%	30%		30%	420

N, nitrogen level; M, PCU application modes; PCU, polymer-coated urea (N = 44.5%); U, urea; CN, conventional N level (540 kg hm^-2^ N per year); RN, reduced N level (420 kg hm^-2^ N per year).

Other management in each treatment was carried out according to the conventional management of local farmers over the years. The rice fields were initially flooded (continuous flooding), followed by alternating wet and dry irrigation. Wheat depended solely on rainfall for irrigation. Weeds were controlled using herbicides and manual weeding, while chemical insecticides were applied for pest management. All treatments were uniformly managed.

### 2.3 Sampling

During the rice and wheat seasons, mature plant samples were randomly collected from each plot (10 plants per plot). All samples were dried in an oven at 80°C for 48 hours until reaching a constant mass and then weighed. The grain yield of each plot was estimated. For rice, the grain moisture content was adjusted to 14.5%, and for wheat, it was adjusted to 12%. The effective panicle density was determined by assessing three representative rice hills in each plot. Pre-harvest measurements included: grains per panicle, grain-filling percentage, and 1,000-grain weight. The plant N concentration was measured with Kjeldahl (Douglas et al., 1980). The total N accumulation in the plant was calculated based on the dry matter yield and the plant N concentration. The N recovery efficiency was the difference between the total N of plants with and without N application divided by the amount of nitrogen application.

Soil samples are collected from the soil surface (depth: 0–15 cm) of all plots using the five-point sampling method, after completing a rice-wheat rotation in 2021 and 2022.After passing through a 2mm sieve, the soil was divided into two parts, one part is stored at -4°C and the other part is air-dried. The soil pH was measured using a pH electrode at a soil to water ratio of 1:2.5. The total organic matter (TOM) content was measured using the potassium dichromate volumetric method (Cai et al., 2024). Total N (TN) content was measured by using a Kjeldahl digestion method (Douglas et al.).After soil samples were treated with H_2_SO_4_-H_2_O_2_ mixture, the total phosphorus (TP) and total potassium (TK) were determined by Autoanalyzer 3 (Bran + Luebbe, Hamburg, Germany) and flame atomic spectrophotometry, respectively. The alkali-hydrolyzed nitrogen (AN), available phosphorus (AP), and available potassium (AK) were determined by the diffusion method, the Olsen method and the ammonium acetate extraction flame photometry method, respectively.

After completing the rice-wheat rotation once, the annual income was calculated. Output value (yuan hm^-2^) = rice grain yield × rice price + wheat grain yield × wheat price. Total cost (yuan hm^-2^) = nitrogen fertilizer cost + topdressing labor cost + other costs. Net income (yuan hm^-2^) = output value - total cost. Output/input ratio = net income/total cost. Controlled-release nitrogen fertilizer, urea, labor cost of single topdressing, and other costs (phosphate fertilizer, potassium fertilizer, seeds, pesticides, machinery, labor costs except topdressing, etc.) were calculated according to the average market price. The price of rice grain was 3142.50 yuan t^-1^; the price of wheat grain was 2720.00 yuan t^-1^; the price of PCU was 4000.00 yuan t^-1^; the price of U was 2300.00 yuan t^-1^; the labor cost of single topdressing was 400.00 yuan hm^-2^; other costs were priced at 7000.00 yuan hm^-2^.

### 2.4 Statistical analysis

The statistical analysis was conducted using SPSS v. 20.0 (SPSS Inc., Chicago, USA) for data analysis and Origin v. 2021 for graphical presentation. The experiment was based on a split-plot design, but due to the need for multiple comparisons, a Randomized Block Design (RBD) model ANOVA was used for the analysis. The Least Significant Difference (LSD) test was applied for *post-hoc* comparisons using a pooled error term (MSe). Significant differences in the means were determined using the least significant difference (LSD) test at the 0.05 probability level. Prior to performing ANOVA, normality of the data was checked using the Shapiro-Wilk test, and homogeneity of variance was tested using Levene’s test. If data did not meet these assumptions, appropriate data transformations (such as log transformation) were applied to stabilize variance and achieve normality.

## 3 Results

### 3.1 Annual rice-wheat yield and N recovery efficiency under conventional nitrogen amount

**Figure 2:**
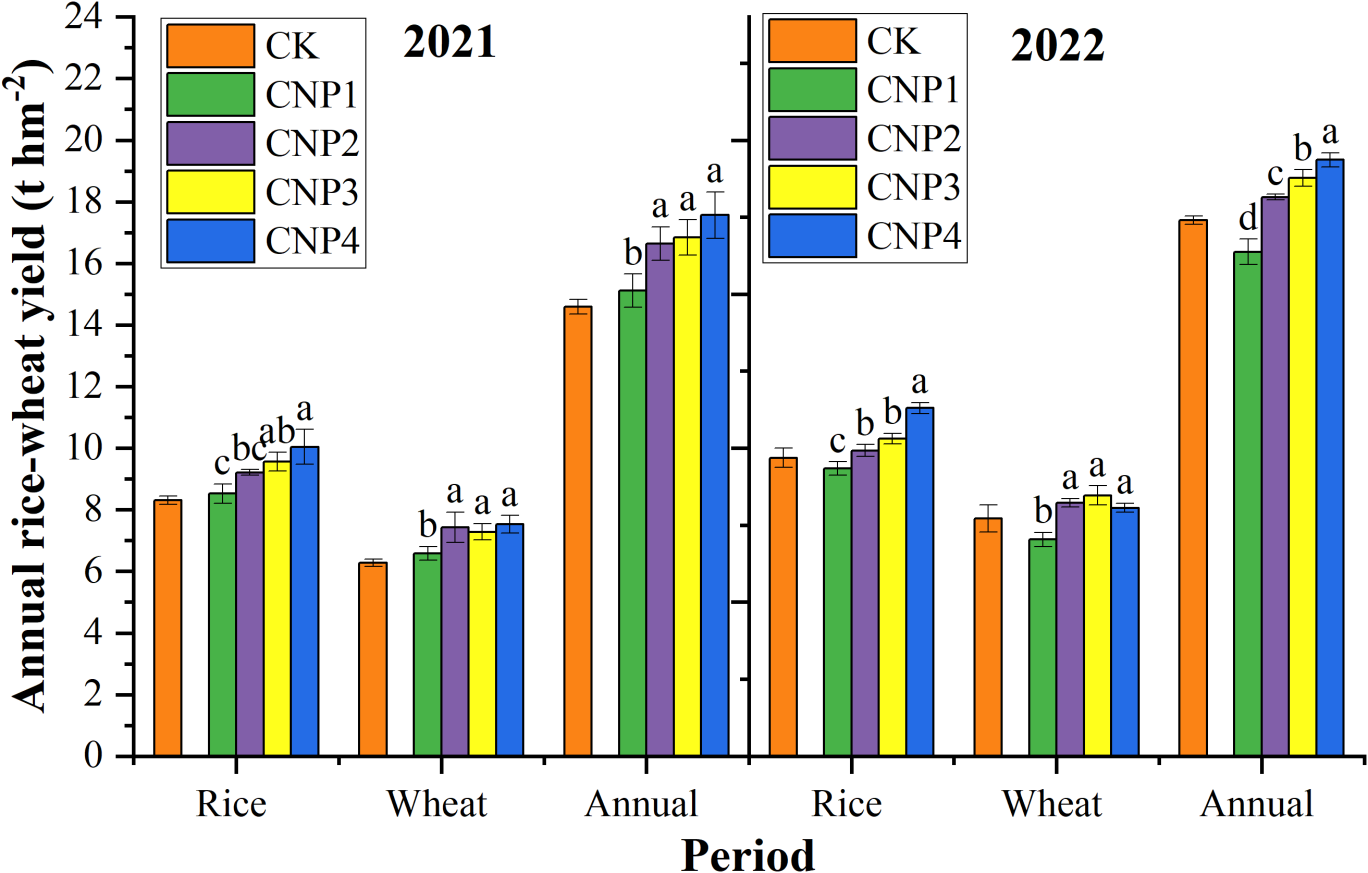
Annual rice-wheat yield under different PCU application modes. CK, Conventional urea application; CN, conventional N level; P1, 100% PCU basal fertilizer application mode; P2, 70% PCU + 30% U basal fertilizer application mode; P3, 70% PCU basal fertilizer + 30% U panicle fertilizer application mode; P4, 40% PCU + 30% U basal fertilizer and 30% U panicle fertilizer application mode. Different letters denote significant differences among different PCU treatments in rice, wheat, and annual rice-wheat rotation at P < 0.05 using the LSD test.

There were significant differences in the annual yield of the rice -wheat rotation under different PCU modes (**Figure 2**). In 2021, CNP1 (100% PCU basal) showed comparable yields to CK, but 2022 wheat yields declined by 5.95%, reducing productivity. This fluctuation suggests that the one-time application of PCU may not sustain long-term yield benefits.

In contrast, the combined PCU + U application modes (CNP2, CNP3, and CNP4) consistently outperformed CK, demonstrating significant yield improvements: annual rice-wheat yields increased by 9.16%, 11.65%, and 15.82%; rice yields by 6.60%, 10.72%, and 18.70%; and wheat yields by 12.49%, 12.85%, and 12.19%, respectively. These results highlight the effectiveness of combined PCU and U applications in promoting crop productivity.

Among PCU + U combinations, the 40% PCU + 30% U basal fertilizer + 30% U panicle fertilizer application treatment produced the highest system yield. This resulted primarily from an 18.7% rice yield increase over CK, demonstrating that balanced N release timing (PCU for sustained supply + U for immediate availability) optimizes nutrient use and enhances productivity in rice-wheat rotations.

**Figure 3:**
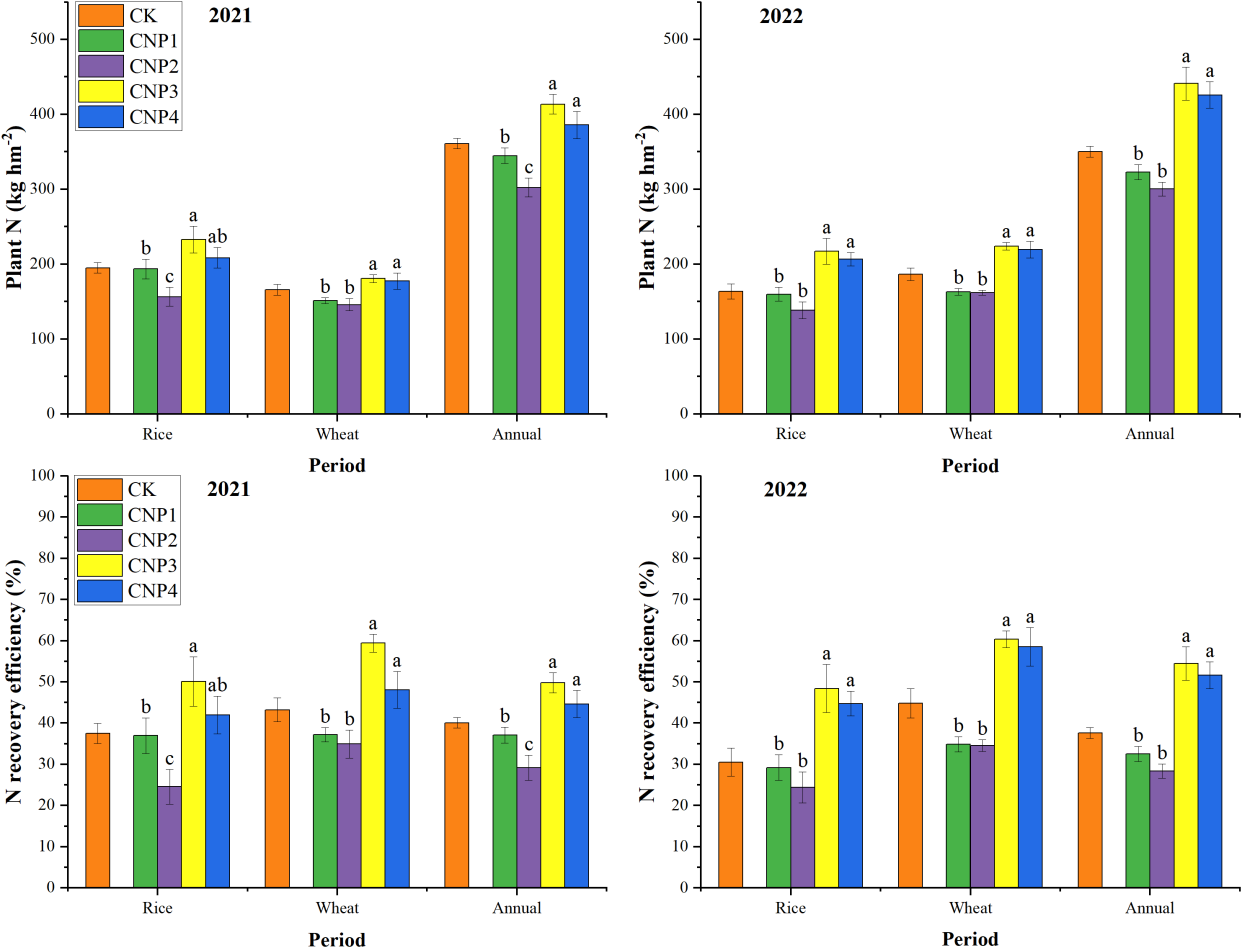
Plant N and N recovery efficiency under different PCU application modes. CK, Conventional urea application; CN, conventional N level; P1, 100% PCU basal fertilizer application mode; P2, 70% PCU + 30% U basal fertilizer application mode; P3, 70% PCU basal fertilizer + 30% U panicle fertilizer application mode; P4, 40% PCU + 30% U basal fertilizer and 30% U panicle fertilizer application mode. Different letters denote significant differences among PCU different treatments in rice, wheat, and annual rice-wheat rotation at P < 0.05 using the LSD test.

Under the same nitrogen application amount, the rice, wheat, and annual plant N in different PCU application modes were basically the same over two years, with the order of CNP3 > CNP4 > CK > CNP1 > CNP2 (**Figure 3**). This indicates that that the application of 100% PCU is not conducive to the absorption and utilization of nitrogen. In contrast, the treatments of PCU + U panicle fertilizer (P3 and P4) showed significantly higher nitrogen uptake than CK, indicating that the combined application of panicle fertilizer on the basis of controlled-release fertilizer can significantly promote the absorption and utilization of nitrogen in the later stage of crops, which is more conducive to the transformation into yield. Since the total nitrogen application levels was consistent throughout the entire rice-wheat growth cycle (300 kg hm^-2^ for rice + 240 kg hm^-2^ for wheat), the N recovery efficiency showed the same trend as plant N.

### 3.2 Annual rice-wheat yield and N recovery efficiency under different reduced nitrogen

ANOVA revealed significant main effects of PCU modes (p<0.01) and nitrogen reduction (p < 0.01) on annual rice-wheat yield, but no significant interaction effect (p > 0.05; **Table 2**). Specifically, nitrogen reduction led to a notable decrease in yield by 5.15%, reflecting the importance of adequate nitrogen supply for maintaining optimal crop productivity. The patterns of different PCU application methods under reduced nitrogen were consistent with those under normal nitrogen fertilization. Despite nitrogen reduction, except for the 100% PCU basal fertilizer treatment, the yield of other PCU treatments was still higher than CK by 7.03-17.05%, demonstrating that even with reduced nitrogen input, PCU can improve nitrogen use efficiency and sustain yield levels. Among the treatments under reduced nitrogen fertilizer, the yield of 40% PCU + 30% U basal fertilizer and 30% U panicle fertilizer treatment was the highest. This suggests that a balanced use of both PCU and urea can optimize nitrogen availability throughout the growth stages, thereby maximizing yield under nitrogen-limited conditions.

**Table 2:** Annual rice-wheat yield under different PCU application modes under nitrogen reduction.

Treatments			Rice yield (t·ha^−1^)		Wheat yield (t·ha^−1^)		Annual rice-wheat yield (t·ha^−1^)		Difference with CK (%)
N	M		2021	2022	2021	2022	2021	2022	
CN	P1		8.53 de	9.33 d	6.59 bc	7.04 b	15.11 de	16.37 d	-2.34
	P2		9.21 bc	9.92 b	7.43 a	8.23 a	16.64 b	18.15 bc	+18.33
	P3		9.56 b	10.31 b	7.28 a	8.46 a	16.84 ab	18.78 ab	+23.31
	P4		10.04 a	11.26 a	7.53 a	8.06 a	17.57 a	19.32 a	+31.64
RN	P1		8.51 de	8.94 d	6.03 c	6.92 b	14.54 e	15.86 d	-9.27
	P2		8.85 cd	9.87 bc	7.41 a	7.78 a	16.26 bc	17.65 c	+12.86
	P3		8.28 e	9.39 cd	7.22 a	8.16 a	15.50 cd	17.55 c	+7.03
	P4		9.16 bc	10.27 b	7.16 ab	8.04 a	16.32 bc	18.31 b	+17.05
Main Plot		N	34.30^**^	25.00^**^	3.53	1.78	19.37**	28.09**	
Sub Plot		M	17.09^**^	31.70^**^	14.74^**^	12.69**	20.19**	56.83**	
		N*M	6.69^**^	3.61^*^	0.88	0.33	1.42	1.42	
		LSD	0.46	0.51	0.58	0.71	0.87	0.66	

N, nitrogen level; M, PCU application modes; N*M, interaction between nitrogen fertilizer level and PCU application modes; CK, Conventional urea application; CN, conventional N level; RN, reduced N level. P1, 100% PCU basal fertilizer application mode; P2, 70% PCU+30% U basal fertilizer application mode; P3, 70% PCU basal fertilizer +30% U panicle fertilizer application mode; P4, 40% PCU+30% U basal fertilizer and 30% U panicle fertilizer application mode. Different lowercase letters indicate significant differences among PCU application modes (p < 0.05). Different uppercase letters indicate significant differences among nitrogen fertilizer levels (p < 0.05). The LSD value is based on the significance level (α = 0.05) under the interaction effect. ^*^significant at P < 0.05, and ^**^significant at P < 0.01.

Annual rice-wheat yield is composed of rice yield and wheat yield. For rice, ANOVA revealed significant (p < 0.05) or highly significant (p < 0.01) effects of nitrogen reduction (RN), PCU application modes, and their interaction on yield (**Table 2**). After nitrogen fertilizer reduction, rice yield under all PCU treatments decreased. Among different PCU application modes, under CN, rice yields ranked: P4 > P3 > P2 > P1 (**Figure 2**). Under RN, the ranking shifted to P4 > P2 > P3 > P1 (**Table 2**). Relative to CN, RN reduced rice yields by 2.24% (P1), 2.19% (P2), 11.19% (P3), and 8.96% (P4) (**Table 2**). Among them, the treatment of 70% PCU basal fertilizer + 30% U panicle fertilizer decreased the most, and the treatment of 70% PCU + 30% U basal fertilizer decreased the least. These results suggest that late-season N applications (panicle stage) are critical for rice productivity, as yield losses under RN were more severe when N was primarily applied late (P3) versus early (P2). Therefore, PCU+U basal was more conducive to stable yield during nitrogen reduction.

In terms of rice yield composition (**Table 3**), reduced nitrogen had a significant effect on effective panicle density, 1000-grain weight, but did not show a strong effect on grains per panicle. PCU application modes significantly influenced all measured parameters (p < 0.05), with particularly strong effects on grains per panicle, grain filling percentage, and 1000-grain weight (p < 0.01). The interaction between reduced nitrogen and PCU application modes had a significant impact on 1000-grain weight (p < 0.01). Nitrogen reduction significantly decreased the number of effective panicles, while increasing the grain filling rate and 1000-grain weight. Under both nitrogen fertilizer levels, the application of U panicle fertilizer reduced the number of effective panicles but increased the number of grains per panicle, grain filling rate, and 1000-grain weight, compared to treatments without U panicle fertilizer. Urea fertilizer proved to be more beneficial for grain filling and yield improvement. Under different PCU application modes combined with nitrogen reduction, there was a significant difference in the 1000-grain weight of rice. The 1000-grain weight increased with the 70% base fertilizer + 30% panicle fertilizer treatment, which led to a decrease in overall yield. The results indicate that U panicle fertilizer had a greater effect on 1000-grain weight. However, increasing yield through the application of fast-release panicle fertilizer was not conducive to maintaining stable yields under nitrogen reduction conditions.

**Table 3:** Rice yield components in different PCU application modes under nitrogen reduction.

Treatments			Effective panicle density	Grains per panicle	Grain filling	1000-grain weight
Year	N	M	(×10^4^ ha^−1^)		(%)	(g)
2021	CN	P1	347.33 b	141.14 bc	91.12 bc	23.62 b
		P2	372.00 a	161.75 ab	87.68 d	22.79 c
		P3	332.00 cd	161.96 a	90.20 c	23.60 b
		P4	341.33 bc	163.03 a	91.00 bc	24.05 a
	RN	P1	326.67 d	137.09 c	92.67 ab	22.86 c
		P2	336.33 bcd	148.64 abc	87.85 d	23.62 b
		P3	312.00 e	156.02 abc	92.32 ab	24.27 a
		P4	328.33 d	159.32 ab	93.73 a	24.21 a
Main Plot		N	73.11^**^	1.93	17.29^**^	9.05^**^
Sub Plot		M	25.62^**^	4.27^*^	27.80^**^	39.83^**^
		N*M	3.34	0.21	1.91	22.42^**^
		LSD	11.02	20.72	1.70	0.32
2022	CN	P1	329.00 ab	136.93 bc	89.69 cd	23.26 a
		P2	348.67 a	138.37 bc	88.55 de	22.26 d
		P3	321.60 b	154.22 a	90.69 bc	22.96 b
		P4	348.60 a	159.12 a	90.54 bc	23.12 ab
	RN	P1	320.93 b	130.91 c	90.88 bc	22.99 b
		P2	331.60 ab	151.31 ab	87.49 e	22.67 c
		P3	320.00 b	144.18 abc	92.44 a	23.14 ab
		P4	326.40 b	146.62 ab	91.71 ab	23.17 a
Main Plot		N	6.12^*^	1.24	5.18^*^	4.63*
Sub Plot		M	3.63^*^	5.49^**^	22.35^**^	58.47**
		N*M	0.86	2.71	3.47^*^	11.33**
		LSD	21.21	15.03	1.43	0.18

N, nitrogen level; M, PCU application modes; N*M, interaction between nitrogen fertilizer level and PCU application modes; CK, Conventional urea application; CN, conventional N level; RN, reduced N level. P1, 100% PCU basal fertilizer application mode; P2, 70% PCU + 30% U basal fertilizer application mode; P3, 70% PCU basal fertilizer + 30% U panicle fertilizer application mode; P4, 40% PCU + 30% U basal fertilizer and 30% U panicle fertilizer application mode. Different lowercase letters indicate significant differences among PCU application modes (p < 0.05). Different uppercase letters indicate significant differences among nitrogen fertilizer levels (p < 0.05). The LSD value is based on the significance level (α = 0.05) under the interaction effect. ^*^significant at P < 0.05, and ^**^significant at P < 0.01.

There were significant differences in wheat yield across different PCU application modes (p < 0.01), but no significant differences were observed with nitrogen reduction or its interaction (p > 0.05; **Table 2**). The wheat yield patterns under different PCU application modes following nitrogen reduction were consistent with those under conventional nitrogen amount (**Figure 3**). From wheat yield components (**Table 4**), both reduced nitrogen and CRU application modes had a significant effect on all three yield components (p < 0.05): No significant RN × PCU interaction was observed for any component (p > 0.05). Nitrogen reduction decreased both the effective panicle and grains per panicle, while increasing the 1000-grain weight. 100% PCU mode was insufficient to meet wheat growth demands. When combined with Urea, the number of effective panicles increased, promoting wheat tillering. The number of effective panicles and 1000-grain weight also increased with urea application, which was beneficial for panicle formation in wheat.

**Table 4:** Wheat yield components in different PCU application modes under nitrogen reduction.

Treatments			Effective panicle density (×10^4^ ha^−1^)	Grains per panicle	1000-grain weight (g)
Year	N	M			
2021	CN	P1	495.00 ab	41.77 bc	40.31 e
		P2	538.33 a	40.45 c	41.12 de
		P3	454.33 b	43.85 a	43.28 ab
		P4	473.67 b	42.62 ab	43.53 a
	RN	P1	471.33 b	38.24 d	42.06 cd
		P2	504.00 ab	38.29 d	42.36 bc
		P3	451.00 b	42.02 b	43.49 a
		P4	470.00 b	42.07 b	43.56 a
Main Plot		N	1.66	36.10^**^	12.70^**^
Sub Plot		M	5.23^*^	26.88^**^	27.06^**^
		N*M	0.37	3.35^*^	3.30
		LSD	54.12	1.44	0.97
2022	CN	P1	594.67 ab	43.10 c	41.36 c
		P2	628.00 a	43.07 c	41.91 bc
		P3	573.33 abc	46.40 a	43.57 a
		P4	578.67 abc	45.80 ab	43.71 a
	RN	P1	522.67 c	39.73 d	41.80 c
		P2	592.67 ab	37.60 d	42.72 b
		P3	517.33 c	45.47 abc	43.86 a
		P4	533.33 bc	43.87 bc	43.92 a
Main Plot		N	13.00^**^	25.02^**^	5.24^*^
Sub Plot		M	4.04^*^	20.97^**^	32.61^**^
		N*M	0.29	2.83	0.49
		LSD	62.06	2.51	0.82

N, nitrogen level; M, PCU application modes; N*M, interaction between nitrogen fertilizer level and PCU application modes; CK, Conventional urea application; CN, conventional N level; RN, reduced N level. P1, 100% PCU basal fertilizer application mode; P2, 70% PCU + 30% U basal fertilizer application mode; P3, 70% PCU basal fertilizer + 30% U panicle fertilizer application mode; P4, 40% PCU + 30% U basal fertilizer and 30% U panicle fertilizer application mode. Different lowercase letters indicate significant differences among PCU application modes (p < 0.05). Different uppercase letters indicate significant differences among nitrogen fertilizer levels (p < 0.05). The LSD value is based on the significance level (α = 0.05) under the interaction effect. ^*^significant at P < 0.05, and ^**^significant at P < 0.01.

The annual rice-wheat N recovery efficiency was significantly different under different PCU application modes and nitrogen reduction (p< 0.05; **Table 5**). Equivalent PCU modes, nitrogen reduction increased the annual rice-wheat N recovery efficiency, especially in wheat. Specifically, among various PCU application modes at the same nitrogen fertilizer level, the annual rice-wheat N recovery efficiency was consistent for both rice and wheat. Notably, the N recovery efficiency of treatment with U panicle fertilizer was significantly higher than that of other treatments, increasing by 47.64%.These results suggest that optimizing nitrogen application methods can effectively enhance nitrogen recovery efficiency in the rice-wheat rotation system.

**Table 5:** Annual rice-wheat N recovery efficiency in different PCU application modes under nitrogen reduction.

Treatment	N	M	Rice		Wheat		Annual rice-wheat NRE (%)
Y			Plant N (kg hm^-2^)	NRE (%)	Plant N (kg hm^-2^)	NRE (%)	
2021	CN	P1	193.51 bc	36.98 c	151.29 bc	37.20 de	37.08 c
		P2	156.36 de	24.60 d	145.88 cd	34.95 e	29.20 d
		P3	232.81 a	50.08 ab	180.63 a	49.43 b	49.79 a
		P4	208.41 b	41.95 c	177.32 a	48.05 bc	44.66 b
	RN	P1	173.69 cd	37.97 c	128.89 e	37.16 de	37.62 c
		P2	144.03 e	25.61 d	138.34 de	42.41 cd	32.81 d
		P3	207.03 b	51.86 a	155.26 bc	51.81 ab	51.84 a
		P4	184.94 c	42.66 bc	163.25 b	56.25 a	48.48 ab
Main Plot		N	15.61^**^	0.37	33.62^**^	9.38^**^	6.44^*^
Sub Plot		M	31.96^**^	33.85^**^	29.41^**^	28.30^**^	81.89^**^
		N*M	0.33	0.02	1.83	1.84	0.60
		LSD	22.09	7.96	12.83	6.30	4.24
2022	CN	P1	159.68 c	29.22 b	162.93 c	34.97 d	32.51 de
		P2	138.28 d	22.08 c	161.94 c	34.56 d	28.37 e
		P3	217.26 a	48.41 a	223.80 a	60.33 ab	54.45 ab
		P4	206.32 ab	44.76 a	219.46 a	58.53 b	51.62 b
	RN	P1	145.88 cd	30.77 b	166.46 c	48.59 c	39.36 c
		P2	135.31 d	26.37 bc	159.09 c	44.49 c	35.09 cd
		P3	193.60 b	50.65 a	198.22 b	66.23 a	58.28 a
		P4	188.84 b	48.67 a	190.44 b	61.91 ab	55.30 ab
Main Plot		N	10.81^**^	3.64	21.52^**^	33.24^**^	24.53^**^
Sub Plot		M	58.04^**^	62.38^**^	82.34^**^	74.35^**^	134.72^**^
		N*M	0.97	0.17	7.80^**^	2.50	0.68
		LSD	18.89	6.74	12.47	6.11	4.56

N, nitrogen level; M, PCU application modes; N*M, interaction between nitrogen fertilizer level and PCU application modes; NRE, nitrogen recovery efficiency; CK, Conventional urea application; CN, conventional N level; RN, reduced N level. P1, 100% PCU basal fertilizer application mode; P2, 70% PCU + 30% U basal fertilizer application mode; P3, 70% PCU basal fertilizer + 30% U panicle fertilizer application mode; P4, 40% PCU + 30% U basal fertilizer and 30% U panicle fertilizer application mode. Different lowercase letters indicate significant differences among PCU application modes (p < 0.05). Different uppercase letters indicate significant differences among nitrogen fertilizer levels (p < 0.05). The LSD value is based on the significance level (α = 0.05) under the interaction effect. ^*^significant at P < 0.05, and ^**^significant at P < 0.01.

### 3.3 Soil fertility

From **Table 6**, it can be seen that for the single factors, TOM, TN, AN, and AK contents had significant or highly significant differences in years, fertilizer modes, and nitrogen levels; AP content had highly significant differences in fertilizer modes and nitrogen levels, pH content had highly significant differences in fertilizer modes, and TK content had highly significant differences in nitrogen levels. In terms of two-factor interactions, TOM, AN and AP contents had highly significant differences in the interactions between year × fertilizer mode, year × nitrogen level, and fertilizer mode ×nitrogen level; pH content had highly significant differences in the interactions between year × nitrogen level and fertilizer mode × nitrogen level; TN content had significant differences in the interactions between year × nitrogen level and year × fertilizer mode. Under the interaction of year × fertilizer mode × nitrogen level, pH, TOM, TN, AP and AK contents showed significant or highly significant differences. These results suggest that different PCU application modes and nitrogen reduction significantly affect soil fertility.

**Table 6:** Analysis of variance for soil fertility with treatments of PCU application modes and nitrogen reduction and their interaction under annual rice-wheat rotation.

Index	Y	N	M	Y*N	Y*M	N*M	Y*N*M
pH	NS	NS	**	NS	*	**	NS
TOM	**	**	**	NS	*	**	NS
TN	**	**	**	NS	NS	NS	NS
TP	NS	NS	NS	*	NS	NS	NS
TK	NS	**	NS	NS	NS	NS	NS
AN	**	**	**	**	**	**	NS
AP	NS	**	**	**	**	**	*
AK	**	**	**	NS	NS	NS	*

Y, year; N, nitrogen level; M, PCU application modes; Y*N, interaction between year and nitrogen fertilizer level; Y*M, interaction between year and PCU application modes; N*M, interaction between nitrogen fertilizer level and PCU application modes; Y*N*M, three factors interaction between year, nitrogen fertilizer level and PCU application mode; TOM, total organic matter; TN, total nitrogen; TP, total phosphorus; TK, total potassium; AN, alkali-hydrolyzed nitrogen; AP, available phosphorus; AK, available potassium. NS, not significant, *significant at P < 0.05, and **significant at P < 0.01.

The experimental data (**Table 7**) revealed a year-on-year decline in soil pH under conventional urea treatment (CK), whereas the PCU application maintained more stable pH levels over the two-year study, demonstrating superior buffering capacity that could enhance agricultural sustainability. Notably, a 25% N reduction had no significant impact on soil pH (p > 0.05). However, within CRU application mode, treatments that received panicle-stage topdressing exhibited higher pH values (with an increase of 2.22%) compared to others. Regarding TOM content, under N reduction, TOM depleted by 1.90-13.58% relative to conventional N application. Among different PCU application modes, the P2 treatment achieved the highest TOM content—reaching 31.5 g/kg (2021) and 30.60 g/kg (2022)—significantly outperforming other treatments (p < 0.01). The P2 treatment still achieved the highest TOM content under nitrogen reduction. Concerning soil TN, a progressive depletion of TN was observed over the years. However, PCU application mitigated this decline, sustaining a 6.79% higher TN content than CK. Crucially, while N reduction generally lowered TN content, the 70% PCU + 30% U basal fertilization strategy showed no statistically significant reduction, suggesting this approach could maintain soil N equilibrium by slowing nutrient release kinetics.

**Table 7:** Soil fertility contents under different PCU application modes in 2021 and 2022.

Treatments			pH	TOM (g/kg)	TN (g/kg)	AN (mg/kg)	AP (mg/kg)	AK (mg/kg)
Year	N	M						
2021		CK	8.03	28.20	1.75	140.9	40.16	103.98
	CN	P1	7.76 c	28.40 b	1.88 bc	141.47 de	30.21 e	101.51 bc
		P2	7.90 b	31.50 a	1.95 a	170.39 a	44.07 a	115.49 a
		P3	7.99 a	30.20 a	1.91 abc	155.09 c	39.73 b	99.86 bc
		P4	8.01 a	27.95 b	1.93 ab	145.08 d	38.42 b	104.63 b
	RN	P1	7.87 b	26.30 c	1.87 c	137.49 ef	35.52 c	101.38 bc
		P2	7.90 b	30.85 a	1.94 a	162.69 b	40.00 b	113.74 a
		P3	8.00 a	26.10 c	1.87 c	135.32 ef	32.47 de	94.41 d
		P4	7.99 a	26.20 c	1.86 c	132.52 f	33.24 cd	99.15 cd
Main Plot		N	2.45	34.96^**^	8.14^**^	57.93^**^	17.41^**^	6.76^*^
Sub Plot		M	25.94^**^	26.76^**^	6.33^**^	81.01^**^	32.76^**^	37.43^**^
		N*M	2.82	3.92*	1.18	5.56*	17.21^**^	1.19
		LSD	0.07	1.56	0.05	6.20	2.88	5.29
2022		CK	7.87	27.0	1.73	133.4	39.19	104.90
	CN	P1	7.76 d	25.68 cd	1.84 bc	133.24 c	27.51 d	107.45 b
		P2	7.86 bc	30.60 a	1.89 a	143.24 a	53.41 a	121.86 a
		P3	8.04 a	29.85 a	1.88 a	128.73 d	47.71 b	103.94 bc
		P4	8.09 a	26.80 bc	1.86 b	124.83 de	40.89 c	105.37 b
	RN	P1	7.88 b	24.90 d	1.82 c	137.72 b	31.04 d	98.05 c
		P2	7.77 cd	27.80 b	1.89 a	143.60 a	37.36 c	118.68 a
		P3	8.00 a	26.10 cd	1.86 b	127.39 d	29.91 d	106.45 b
		P4	8.01 a	26.20 cd	1.85 b	122.53 e	32.01 d	103.47 bc
Main Plot		N	0.84	0.84	29.14^**^	6.20^*^	0.08	62.39^**^
Sub Plot		M	30.27^**^	30.27^**^	21.55^**^	22.67^**^	71.17^**^	28.71^**^
		N*M	4.66^*^	4.41^*^	2.12	2.09	15.24^**^	2.19
		LSD	0.10	1.58	0.02	4.41	5.32	7.13

N, nitrogen level; M, PCU application modes; N*M, interaction between nitrogen fertilizer level and PCU application modes; TOM, total organic matter; TN, total nitrogen; AN, alkali-hydrolyzed nitrogen; AP, available phosphorus; AK, available potassium. CK, Conventional urea application; CN, conventional N level; RN, reduced N level. P1, 100% PCU basal fertilizer application mode; P2, 70% PCU + 30% U basal fertilizer application mode; P3, 70% PCU basal fertilizer + 30% U panicle fertilizer application mode; P4, 40% PCU + 30% U basal fertilizer and 30% U panicle fertilizer application mode. Different lowercase letters indicate significant differences among PCU application modes (p < 0.05). Different uppercase letters indicate significant differences among nitrogen fertilizer levels (p < 0.05). The LSD value is based on the significance level (α = 0.05) under the interaction effect. ^*^significant at P < 0.05, and ^**^significant at P < 0.01.

In terms of the soil AN content, the treatment of 70% PCU + 30% U basal fertilizer was significantly higher than other treatments under both conventional and reduced nitrogen levels. For soil AP content, the CK treatment showed a decrease over the years. Under conventional nitrogen levels, AP content in PCU application treatments generally increased, except for the 100% PCU treatment. In contrast, AP content in nitrogen reduction treatments decreased, with the RNP2 treatment exhibiting the highest AP content. Regarding soil AK content, the 70% PCU + 30% U basal fertilizer treatment consistently outperformed other treatments.

In conclusion, the choice of fertilizer application modes and nitrogen level play a crucial role in shaping soil fertility. These findings highlight the need to select the right mode of nitrogen reduction to optimize soil health and crop yields.

### 3.4 Correlation studies

From **Table 8**, the rice-wheat yield was significantly positively correlated with plant N, soil pH, and AP, indicating that increasing the soil AP content and plant N could enhance yield. Under PCU application mode, a synergistic relationship exists between yield improvement and the increase in soil pH. Furthermore, plant N recovery efficiency was positively correlated with plant N and soil pH, and negatively correlated with the AN and AK content of soil, which indicated that improving plant N recovery efficiency could increase plant N, while reducing soil active N and K content. The soil TOM content was significantly positively correlated with TN, AN, AP, and AK. This highlights the importance of increasing the levels of active nitrogen, phosphorus, and potassium in the soil during plant growth to enhance soil organic matter content. In summary, optimizing soil AP and plant N, along with managing soil pH, are essential strategies for improving rice-wheat yield and enhancing soil fertility.

**Table 8:** Correlation analysis among each index for crop traits and soil fertility under annual rice-wheat rotation under different treatments with controlled-release urea in 2021 and 2022.

Traits^a^	Annual Yield	Plant N	NRE	pH	TOM	TN	AN	AP	AK
Annual Yield	1								
Plant N	0.544^*^	1							
NRE	0.362	0.844^**^	1						
pH	0.511^*^	0.760^**^	0.795^**^	1					
TOM	0.201	-0.052	-0.342	0.006	1				
TN	0.036	-0.117	-0.317	0.063	0.827^**^	1			
AN	-0.307	-0.481	-0.627^**^	-0.303	0.734^**^	0.822^**^	1		
AP	0.503^*^	0.044	-0.238	0.213	0.769^**^	0.536^*^	0.353	1	
AK	0.377	-0.466	-0.600^*^	-0.361	0.535^*^	0.474	0.432	0.572^*^	1

NRE, nitrogen recovery efficiency; TOM, total organic matter; TN, total nitrogen; AN, alkali-hydrolyzed nitrogen; AP, available phosphorus; AK, available potassium. NS, not significant, *significant at P < 0.05, and **significant at P < 0.01.

## 4 Discussion

### 4.1 Effects of PCU application modes on annual yield and N recovery efficiency

Both controlled-release fertilizers (CRF) and split urea applications represent effective strategies for yield enhancement (Ercoli et al., 2013; Jin et al., 2016). In rice production, Ji et al. (2011) found that a combined application of CRF and urea in a 7:3 ratio resulted in significantly higher yields compared to using either fertilizer type alone. Similarly, wheat studies by Li et al. (2024) revealed that while a single basal application of 100% CRF reduced yields, a combination of 60% urea-formaldehyde with CRF as basal fertilizer followed by 30% urea topdressing substantially increased productivity. The results of our study indicate that, except for a single application of PCU, all other PCU treatments resulted in increased rice, wheat, and their annual yields. This finding aligns with previous studies. Furthermore, among these treatments, 40% PCU + 30% U basal fertilizer and 30% U panicle fertilizer application mode (P4) had the highest overall yield. The application of different fertilizer types at specific growth stages enables more precise nitrogen supply to crops (e.g., during rice tillering stage and wheat jointing stage) (Silvia and Marco, 2021). CRU maintain stable nitrogen availability throughout the growing season through gradual nutrient release, preventing short-term excess. In contrast, U provides immediate nitrogen supplementation during peak crop demand periods. The combined application of CRU and U optimizes crop growth potential.

Studies have demonstrated that the exclusive application of CRU may reduce rice yield by decreasing the effective panicle number (Wu et al., 2024). This effect is attributed to the nitrogen (N) release dynamics of polymer-coated urea (PCU), which is influenced by temperature and humidity. As illustrated in **Figure 1**, during the early tillering stage, PCU releases only 40% of total N, significantly lower than the 70% N release from conventional urea. This suboptimal N availability restricts early nitrogen uptake, leading to delayed and insufficient tillering. However, combining PCU with urea (U) as a basal fertilizer enhances early-stage N availability, improving N uptake, leaf area expansion, photosynthetic efficiency, and tillering acceleration, thereby increasing effective panicle formation (Zhang Z. et al., 2022). During the mid-growth stage, PCU ensures continuous N supply, promoting carbohydrate accumulation (total soluble sugars, starch, lignin, and cellulose) in the basal stem’s second internode, which enhances biomass production and lodging resistance (Wu et al., 2024). At the reproductive stage, PCU + U panicle fertilizer provides sustained nutrient availability, can enhance enzyme activities related to nitrogen metabolism in the flag leaves, as well as the oxidase activities and nitrogen uptake in the roots (Wang et al., 2018), which is conducive to the formation of spikelet and increases grains per panicle. At the same time, it also delays leaf senescence, improves the photosynthetic rate, increases photosynthetic products, and improves grain weight during the filling stage (Zhang G. X. et al., 2022; Zhou et al., 2021). In rice, the 1000-grain weight decreased due to the increase in grain number per panicle. However, in wheat, the panicle number changed little, and the increase of panicle fertilizer was also beneficial to increase the 1000-grain weight. In conclusion, the combined application of PCU + U basal and panicle fertilizer optimizes N availability across growth stages, supporting annual rice-wheat yield sustainability.

N use efficiency (NUE), a critical metric reflecting crop nitrogen uptake and utilization, plays a pivotal role in optimizing fertilization strategies and advancing agricultural sustainability. Research by Li et al. (2010) demonstrated that a blended application of 30% U with two types of coated urea (35% with 90-day release and 35% with 120-day release) enhanced wheat nitrogen uptake by 6.11% compared to conventional urea, owing to better synchronization between nitrogen release patterns and crop demand, thereby minimizing losses through leaching and volatilization. In rice, CRU has proven effective in reducing nitrogen loss and improving NUE (Chen et al., 2020), though some studies report reduced late-stage nitrogen uptake due to release-rate mismatches with peak demand periods. This limitation can be mitigated through strategic combinations with conventional urea, as demonstrated (Huang et al., 2024; Wei et al., 2017). Our study demonstrated that combining CRU with U panicle fertilizer (P3 and P4) significantly improves NUE, which aligns with previous research findings. However, when combined with U basal fertilizer (P1 and P3), NUE showed a decreasing trend. The application method of CRU significantly impacts NUE (P < 0.05). This phenomenon may be attributed to the temperature- and humidity-dependent nitrogen release characteristics of CRU, where semi-permeable membranes regulate nitrogen hydrolysis rates (Wang et al., 2016). The gradual nitrogen release from CRU reduces ammonia volatilization and nitrogen leaching, thereby minimizing nitrogen losses and improving NUE. However, climatic factors including precipitation, temperature, and humidity influence both the nitrogen release kinetics and crop nitrogen uptake efficiency. For instance, under humid conditions, accelerated fertilizer dissolution and nitrogen release may enhance nitrogen volatilization while compromising crop absorption. Conversely, arid environments restrict fertilizer release, leading to reduced NUE (Wood et al., 2023). Regarding fertilization modes, CRU treatments without panicle fertilizer supplementation fail to provide adequate nitrogen during grain filling, resulting in lower plant nitrogen content and consequently reduced total nitrogen accumulation and NUE.

### 4.2 Effects of PCU application modes under nitrogen reduction

Reducing excessive fertilization not only prevents environmental pollution but also enhances crop roots’ efficiency in absorbing nitrogen from the soil, thereby improving nitrogen conversion rates (Silvia and Marco, 2021; Wang et al., 2016). Previous studies have shown that nitrogen reduction can improve the nitrogen use efficiency of crops (Li et al., 2010), and treatments with PCU application can achieve the same yield as conventional urea application with a 30% reduction in nitrogen (Qin et al., 2008; Zheng et al., 2016). In this experiment, under reduced nitrogen conditions (20% N reduction in rice, 25% N reduction in wheat), rice-wheat nitrogen use efficiency was improved, rice-wheat yields with PCU + urea treatments, except for 100% PCU, were similar to the control (CK), consistent with previous studies. Compared to treatment CN, annual yields under treatments RN with different PCU application modes decreased, though to varying degrees, with the largest decrease in P3 and the smallest in P2. This suggests that nitrogen fertilizer reduction under late fertilizer application is more detrimental to yield, while early fertilizer application is more conducive to stable yield, especially in rice.

High nitrogen level increased root cytokinin and decreased chrysolactone at tillering stage, and significantly inhibited the expression of FC1/OsTB1, thus increasing the tillering number of rice (Minakuchi et al., 2010). Reducing nitrogen input at tillering stage would lead to less and later tillering, resulting in yield reduction due to less effective panicle number. High panicle nitrogen level can effectively promote panicle differentiation and increase the number of grains per panicle in the early stage (Toshiyuki et al., 2006), and can increase chlorophyll content of crop leaves, increase photosynthetic area and accelerate photosynthetic production in the later stage (Li et al., 2012). Concurrently, it can increase nitrate reductase and accelerate nitrogen absorption and utilization, which is conducive to grain filling, and then increase the setting rate and 1000-grain weight (Li et al., 1998). Reducing nitrogen levels in panicle fertilizer may lead to reduced panicle number and inadequate grain filling, which has a negative impact on yield. Therefore, nitrogen reduction in the early and late growth period has a great impact on yield.

According to the path analysis in **Table 9**, the path coefficient of grain number per panicle is the smallest and the effective panicle density is the largest. Therefore, it can be seen that relying on a large amount of panicle fertilizer to increase grain number per panicle and thus increase yield is not conducive to maintaining yield during nitrogen reduction. Consequently, nitrogen reduction combined with base fertilizer is more conducive to stable yield. In wheat, the path coefficient of grain number per panicle is the smallest, the effective panicle number and 1000-grain weight are larger, so the effect of nitrogen reduction in the early and late stages is not significant.

**Table 9:** Correlation coefficients of rice and wheat yield and its components.

Crops	Rice		Wheat	
	Simple correlation coefficient	Direct path coefficient	Simple correlation coefficient	Direct path coefficient
Effective panicle density	0.207	0.142	0.422	0.442
Grains per panicle	0.204	0.465	0.49	0.258
Grain filling	-0.159	0.143	—	—
1000-grain weight	-0.341	0.581	0.443	0.392

**Figure 4:**
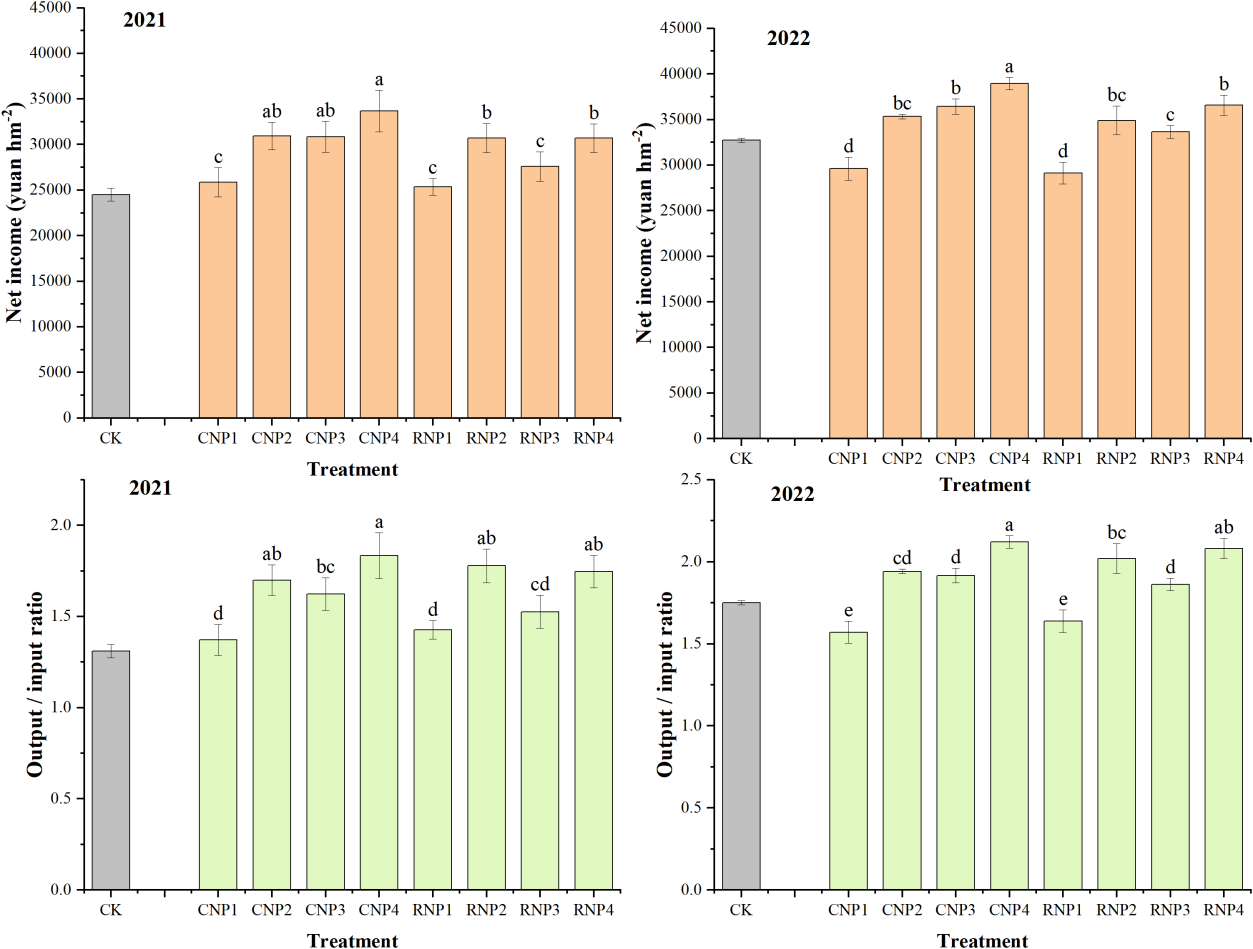
Annual rice-wheat net income and Rice-wheat output/input ratio under different PCU application modes. CK, Conventional urea application; CN, conventional N level; RN, reduced N level. P1, 100% PCU basal fertilizer application mode; P2, 70% PCU + 30% U basal fertilizer application mode; P3, 70% PCU basal fertilizer + 30% U panicle fertilizer application mode; P4, 40% PCU + 30% U basal fertilizer and 30% U panicle fertilizer application mode. Different letters denote significant differences among different PCU treatments at P < 0.05 using the LSD test.

From an economic perspective, while CRU tends to have a higher initial cost compared to urea, its slow-release nature can result in reduced fertilizer usage over time, as fewer applications are needed. This can lead to cost savings in the long run, especially in large-scale agricultural systems. Moreover, by reducing nitrogen losses to the environment, CRU can contribute to more sustainable agricultural practices, lowering the risk of water pollution and greenhouse gas emissions associated with fertilizer use. From the net income and output/input (**Figure 4**), we can see that PCU + U had a higher the output/input than CK, both under CN and RN. Under the conventional nitrogen level, the P4 treatment is the highest. Under reduced nitrogen level, the output/input ratio of the treatment with large nitrogen input in the early stage increased, while the output/input ratio of the treatment with large nitrogen input in the later stage decreased. Specifically, the P2 treatment showed a strong performance after nitrogen reduction, demonstrating a better balance between reducing nitrogen fertilizer input and overall economic benefits. This was because reducing nitrogen and one-time fertilization saved labor and time, while mixing urea with CRU maintained yield. The application of CRU, coupled with effective nutrient management practices, may thus provide both economic and environmental benefits, making it a more sustainable choice for long-term crop production.

### 4.3 Effects of PCU application modes on soil fertility

The enhancement of soil fertility represents a pivotal aspect of improving soil quality, thereby facilitating the advancement of sustainable agriculture (Johnston et al., 2009). Ladha et al. (2003) reported that long-term urea application increased organic matter and nitrogen content but led to a continuous decline in rice yield due to insufficient nutrient supply and soil acidification. Geng et al. (2015) conducted a seven-year rice-rape rotation test, while Zheng et al. (2016) conducted a five-year wheat-corn rotation test. Both studies found that long-term application of CRU significantly reduced soil acidification and increased TOC and TN content in soil compared with U. PCU application, especially when constituting 70% of total nitrogen fertilizer, increased TOC and active N, P, and K content in the soil compared to U. This is consistent with previous generations. The possible reason is that CRU has less nitrogen loss in the field than urea (Kenawy et al., 2020), resulting in higher nitrogen content retained in the soil. Secondly, CRU basal application provides a continuous release of nitrogen fertilizer, which is conducive to the growth of crop roots and stimulates the roots to secrete more root exudates to dissolve insoluble salts in the soil, thus increasing the content of available nutrients in the soil. Finally, the CRU obtained higher yield and biomass, and the stubble decomposed in the soil, thereby increasing the organic matter content.

Additionally, the reduction in soil acidification with CRU application, as observed in two-year study, had broader implications for soil sustainability. Soil pH is a critical factor that governs nutrient availability; when soil becomes too acidic, nutrients such as calcium and magnesium become less available, which can hinder crop growth. By reducing soil acidification, CRU helps maintain a more favorable soil pH over time, thereby improving nutrient availability and enhancing microbial activity. This creates a more sustainable growing environment, benefiting soil health and improving crop productivity in the long run.

Under reduced nitrogen, the 70%PCU + 30%U basal fertilizer application mode demonstrated optimal effectiveness in maintaining higher post-harvest soil nutrient availability, while the PCU + U panicle fertilizer showed the most significant reduction in soil nutrient content. This phenomenon may be attributed to urea application at the 3^rd^ last leaf stage stimulating vigorous growth in both aboveground and belowground plant parts, with crop development showing strong correlations with soil nutrient status, as dry matter accumulation increased, so did nutrient uptake from the soil. Furthermore, urea application during panicle initiation enhanced root activity, exudate production and overall root system development, thereby promoting more efficient plant uptake of available soil nutrients and consequently (Ming Qian Zhang et al., 2013) leading to greater depletion of soil nutrient reserves.

## 5 Conclusions

Compared to the sole application of controlled-release fertilizer or urea, the combined application significantly enhances the annual rice-wheat yield. Specifically, PCU + U basal fertilizer application modes increase yield by boosting the number of effective panicles, while PCU + U panicle fertilizer application modes increase yield by improving grain number per panicle and nitrogen use efficiency. Additionally, the combined application enhances soil nutrient availability, with PCU + U basal fertilizer application modes being particularly effective in improving soil quality. Under reduced nitrogen conditions, 70% PCU + 30% U basal fertilizer application mode stabilizes annual yields by ensuring effective panicle formation early, demonstrating strong potential for broader application in nitrogen reduction practices.

## Data Availability

The raw data supporting the conclusions of this article will be made available by the authors, without undue reservation.
